# Functional Fragments of AIMP1-Derived Peptide (AdP) and Optimized Hydrosol for Their Topical Deposition by Box-Behnken Design

**DOI:** 10.3390/molecules24101967

**Published:** 2019-05-22

**Authors:** Jeong-Jun Lee, Young-Min Han, Tae-Wan Kwon, Dong Hyun Kim, Han Sol Lee, Woo Jin Jung, Jina Kim, Sujin Kang, Sang Kyum Kim, Cheong-Weon Cho, Keong-Ryoon Lee, Dae-Duk Kim, Min Chul Park, Jae-Young Lee

**Affiliations:** 1 College of Pharmacy, Chungnam National University, Daejeon 34134, Korea; daegeon0705@cnu.ac.kr (J.-J.L.); gksdudals93@naver.com (Y.-M.H.); mds_wantae@naver.com (T.-W.K.); gcy70426@gmail.com (D.H.K.); sol4273@naver.com (H.S.L.); woojin@cnu.ac.kr (W.J.J.); sangkim@cnu.ac.kr (S.K.K.); chocw@cnu.ac.kr (C.-W.C.); 2 CureBio Research Center, Suwon 16229, Korea; jnkim@curebio.co.kr (J.K.); sjkang@curebio.co.kr (S.K.); 3 Laboratory Animal Resource Center, Korea Research Institute of Bioscience and Biotechnology, Ochang 28116, Korea; kyeongrlee@kribb.re.kr; 4 College of Pharmacy and Research Institute of Pharmaceutical Sciences, Seoul National University, Seoul 08826, Korea; ddkim@snu.ac.kr

**Keywords:** AIMP1-derived peptide (AdP), cosmeceutical peptide, Box-Behnken design, hydrosol, topical delivery of peptides

## Abstract

Aminoacyl-tRNA synthetase complex-interacting multifunctional protein 1 (AIMP1)-derived peptide (AdP) has been developed as a cosmeceutical ingredient for skin anti-aging given its fibroblast-activating (FA) and melanocyte-inhibiting (MI) functions. However, a suitable strategy for the topical delivery of AdP was required due to its low-permeable properties. In this study, FA and MI domains of AdP (FA-AdP and MI-AdP, respectively) were determined by functional domain mapping, where the activities of several fragments of AdP on fibroblast and melanocyte were tested, and a hydrosol-based topical delivery system for these AdP fragments was prepared. The excipient composition of the hydrosol was optimized to maximize the viscosity and drying rate by using Box-Behnken design. The artificial skin deposition of FA-AdP-loaded hydrosol was evaluated using Keshary-Chien diffusion cells equipped with Strat-M membrane (STM). The quantification of the fluorescent dye-tagged FA-AdP in STM was carried out by near-infrared fluorescence imaging. The optimized hydrosol showed 127-fold higher peptide deposition in STM than free FA-AdP (*p* < 0.05). This work suggests that FA- and MI-AdP are active-domains for anti-wrinkle and whitening activities, respectively, and the hydrosol could be used as a promising cosmetic formulation for the delivery of AdPs to the skin.

## 1. Introduction

Skin aging, characterized by the formation of wrinkles and mottled pigmentation and the roughness and dryness of the skin, is a complicated biological process, caused by various intrinsic and extrinsic factors [1,2]. Extracellular matrix (ECM), a non-cellular structural component consisting of various biomacromolecules, is closely associated with the aging process of the skin [3,4,5]. Collagen, the main component of skin ECM, is steadily fragmented and decreased in the skin by aging [6,7]. The fragmented collagen compromises the structural integrity and metabolic function of the skin, inducing wrinkles [8,9]. Excessive melanin deposition in the epidermis is another major trait of skin aging, resulting in skin pigmentation. This process is caused by the undesirable activation of melanocytes under UV irradiation [10,11]. Thus, cosmeceutical ingredients for skin anti-aging have been developed focusing on the reduction of wrinkles and pigmentation of the skin. Recently, various peptides derived from cytokines and growth factors became the center of attention in the cosmeceutical field due to their anti-aging activities [6,12].

Aminoacyl-tRNA synthetase complex-interacting multifunctional proteins (AIMPs) are known to form the multi-synthetases complex (MSC) with aminoacyl-tRNA synthetases (ARSs). This MSC functions as crucial components of the protein translation machinery [13]. Among the members of this protein family, AIMP1 has garnered much attention in the cosmeceutical field given its wound-healing activity [14,15]. The secreted AIMP1 induces collagen synthesis of fibroblasts in the wound lesion via the ERK-dependent pathway, which leads to rapid post-trauma repairing [16]. Moreover, AIMP1 stimulates mesenchymal stem cells via protein kinase B (PKB)-mediated activation of β-catenin and downstream interactions, increasing the level of MSCs in the bloodstream [17]. Interestingly, these diverse extracellular functions of AIMP1 are attributed to the different regions on its structure. For example, this protein promotes the proliferation of fibroblasts through its 6–46 amino acid region [18]. Thus, AIMP1-derived peptide (AdP; INCI name: sh-oligopeptide 5/sh-oligopeptide 5 SP) of this region was developed as a cosmeceutical for skin anti-aging. According to the previous study, AdP has dual functions, namely fibroblast-activating and melanocyte-inhibiting activities [13]. However, no information on the active domains for these functions has been reported.

The delivery of peptides through the skin is challenging due to the protective nature of the skin. The physiological role of the skin is a barrier against environmental factors like UV light, microorganisms, and xenobiotics [19]. Stratum corneum (SC) is the outermost layer of the skin and composed of crystalline lipid matrix embedded with corneocytes [20]. Underneath the SC, the viable epidermis consisting of stratum granulosum, stratum spinosum, and stratum basale gives structural integrity of the skin. Among these layers, the SC is the main hurdle for peptide delivery, as it is composed of a highly hydrophobic lipid matrix and closely packed corneocytes. 

Several attempts have been made to overcome this issue, which includes liposomes, microemulsion, iontophoresis, electroporation, and microneedle [21,22,23,24,25]. However, the most reliable and reproducible approach for improving skin delivery uses penetration enhancers [26]. Ethanol, a representative penetration enhancer for peptide, was adopted in this study [27]. In addition, sodium carboxymethylcellulose (CMC), a non-toxic thickening agent, was selected to prepare the hydrosol-based topical delivery system. CMC is the most commonly used cellulose derivative today, of which applications include cosmetics, food additives, paper, textile, and pharmaceutical products [28]. CMC is produced by the carboxymethylation of polysaccharides, such as starch, cotton linters, and cashew tree gum, which renders water soluble, chemically stable, non-toxic, biocompatible, and biodegradable properties [29]. 

The composition of the hydrosol was optimized by using the response surface methodology (RSM) to secure appropriate viscosity and drying rate for dermal application. This statistical technique requires much less experimentation and time to achieve critical quality attributes of the product compared with one-factor-at-a-time (OFAT) methods [30,31]. RSM also provides the significance of the independent variables and their interactions [32]. The Box-Behnken design (BBD) used in this study belongs to RSM and has many advantages compared with the other RSM designs, requiring fewer runs in the three- or four-variable case [33]. Moreover, BBD can avoid combined factor extremes, as the data at the midpoint of the edge are used to construct the model [34]. 

In this study, the fibroblast-activating and melanocyte-inhibiting domains of AdP (FA- and MI-AdP, respectively) were determined to address functional separation. FA-AdP fragment was loaded to the optimized hydrosol, and its artificial skin deposition was evaluated using near-infrared fluorescence imaging technique. 

## 2. Results

### 2.1. Functional Fragments of AIMP1-Derived Peptide (AdP)

**Figure 1:**
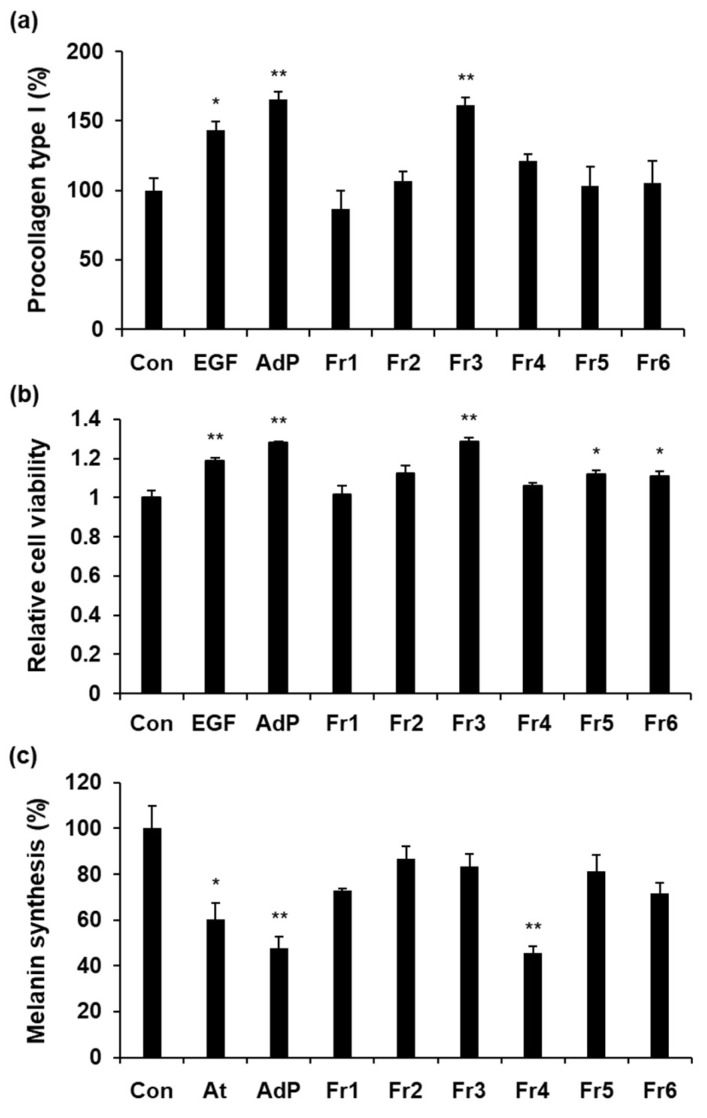
Fibroblast-activating (FA) and melanin-inhibiting (MI) domain mapping of AIMP1-derived peptide (AdP): (**a**) Foreskin fibroblasts were treated with EGF (1 μg/mL), AdP (0.1 μg/mL), and fragments of AdP (Fr1–Fr6; 0.1 μg/mL). After 24 h incubation, procollagen type I was determined in cultured media by ELISA; (**b**) after 48 h incubation, CCK8-assay was performed to monitor the proliferation of foreskin fibroblasts; (**c**) melanocytes, pre-treated with α-MSH (0.5 μM), were treated with albutin (At, 10 μM), AdP (1 μg/mL) and fragments of AdP (Fr1–Fr6, 1 μg/mL). After 48 h incubation, intracellular melanin was measured. Data are presented as the mean ± standard deviation (SD) (*n* = 3). * *p* < 0.05 and ** *p* < 0.01, significantly different from the control.

AdP, 6- to 46-residue fragment of AIMP1, has dual functions (i.e., fibroblast-activating and melanocyte-inhibiting activities) in the skin-associated cell. For functional separation, fragments of AdP (Fr1: 6–20, Fr2: 10–24, Fr3: 14–28, Fr4: 18–32, Fr5: 26–40, and Fr6: 32–46 residue fragment of AIMP1) were produced by solid-phase synthesis and tested on fibroblasts and melanocytes. To investigate the fibroblast-activating domain of AdP (FA-AdP), collagen induction and cell proliferation were determined after the treatment of each fragment. Fr3 showed similar induction activities with AdP on collagen synthesis and fibroblast proliferation (Figure 1a,b). Compared with EGF (1 μg/mL), Fr3 (0.1 μg/mL) exhibited 15% and 10% more potent activities for collagen induction and fibroblast proliferation, respectively. To identify the melanocyte-inhibiting domain of AdP (MI-AdP), the fragments of AdP (Fr1–6) were treated to α-MSH induced melanocytes. Among them, Fr4 showed comparable inhibition of melanin synthesis induced by α-MSH with that of AdP (Figure 1c). Moreover, compared with albutin (10 μM), Fr4 (1 μg/mL) exerted 35% higher inhibition effect on melanin synthesis.

### 2.2. Cytotoxicity and Immunogenicity Tests of FA- and MI-AdP

**Figure 2:**
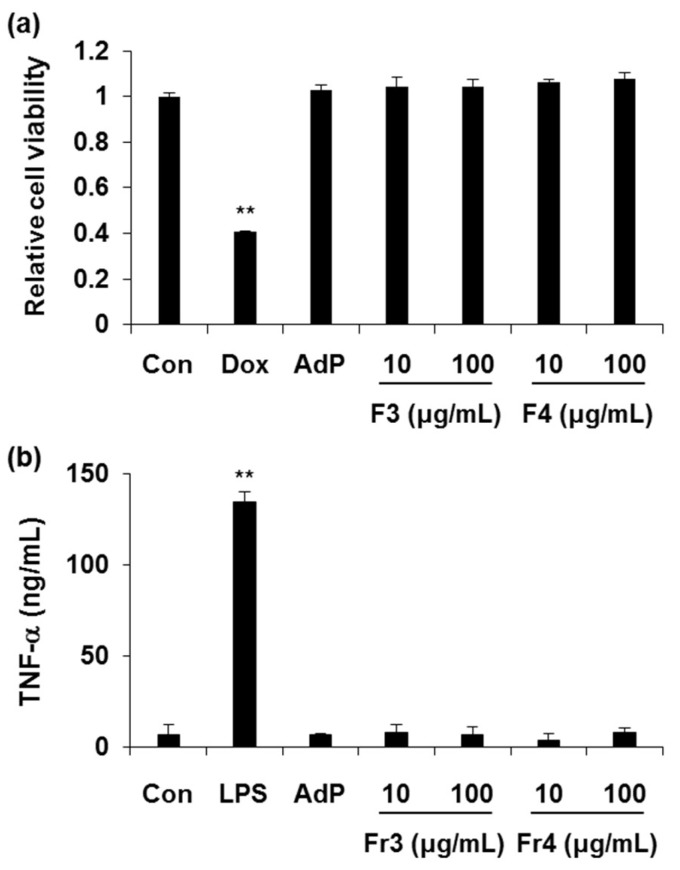
Non-cytotoxicity and non-immunogenicity of FA-AdP and MI-AdP: (**a**) To test cytotoxicity of FA-AdP and MI-AdP, keratinocytes (HaCaT cells) were incubated with doxorubicin (Dox, 10 μM), AdP (100 μg/mL), FA-AdP (10 or 100 μg/mL), and MI-AdP (10 or 100 μg/mL). After 24 h incubation, cytotoxicity was determined by CCK-8 assay; (**b**) for the immunogenicity test, monocytes (THP-1 cells) were used. After 12 h incubation with LPS (50 ng/mL), AdP (100 μg/mL), FA-AdP (10 or 100 μg/mL), and MI-AdP (10 or 100 μg/mL), the TNF-α level was measured in cultured media by ELISA. Data are presented as the mean ± SD (*n* = 3). ** *p* < 0.01, significantly different from the others.

Since safety issue is important in the cosmeceutical filed, cytotoxicity and immunogenicity tests were performed on FA-AdP and MI-AdP (i.e., Fr3 and Fr4, respectively). To guarantee the safety of these peptides, a high amount of FA-AdP or MI-AdP was treated to achieve 100 × the effective concentration. In keratinocytes, FA-AdP and MI-AdP showed no cytotoxicity up to 100 μg/mL (Figure 2a). For immunogenicity tests, FA-AdP and MI-AdP were treated to monocytes. As shown in Figure 2b, the TNF-α level of peptide-treated groups exhibited no significant difference compared with that of the control group (Figure 2b).

### 2.3. Viscosity of AdP Hydrosol

To demonstrate the improved topical delivery of AdP through stratum corneum (SC), FA-AdP-loaded hydrosol formulations consisting of CMC, ethanol, and glycerin were prepared and evaluated. The viscosity values of the AdP hydrosols ranged from 116.1–267.1 cps (Table 1). 

The reduced mathematical model equation for viscosity (*y*_1_) in uncoded units is as below:

*y*_1_ = 69.6 + 282.5·*x*_1_ + 0.669·*x*_2_ + 0.09·*x*_3_ + 0.0976·*x*_2_·*x*_3_ (1)

where each term exhibited a significant effect on the viscosity of the hydrosol (*p* < 0.05; Table 2). 

**Figure 3:**
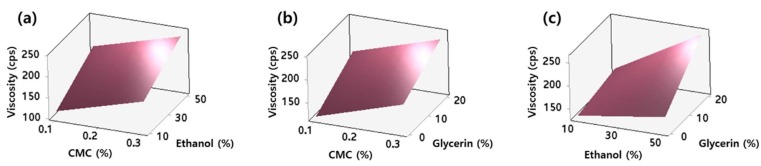
Response surface plots showing the effect of (**a**) CMC (*x*_1_) and ethanol (*x*_2_); (**b**) CMC (*x*_1_) and glycerin (*x*_3_); and (**c**) ethanol (*x*_2_) and glycerin (*x*_3_) on viscosity (*y*_1_). The hold values of CMC, ethanol, and glycerin were set at 0.2%, 30%, and 10%, respectively.

The viscosity values increased as the concentrations of CMC (*x*_1_), ethanol (*x*_2_), and glycerin (*x*_3_) changed from low to high values. The coefficients of *x*_1_, *x*_2_, *x*_3_, and *x*_2_·*x*_3_ in the coded regression equation were 28.25, 32.91, 30.16, and 19.53, respectively, indicating the linear terms have more substantial impacts on the viscosity than the interaction term. The actual, adjusted, and predicted coefficient of determination (*R*^2^) values were 0.9049, 0.8668, and 0.7364, respectively. The similarity among these values suggests that the model was not over-fitted. The response surface plots showing the effect of the independent variables on the viscosity of the hydrosol are presented in Figure 3. As expected from the equation mentioned above, the concentrations of CMC, ethanol, and glycerin exhibited positive effects on viscosity. 

### 2.4. Drying Rate of AdP Hydrosol

**Figure 4:**
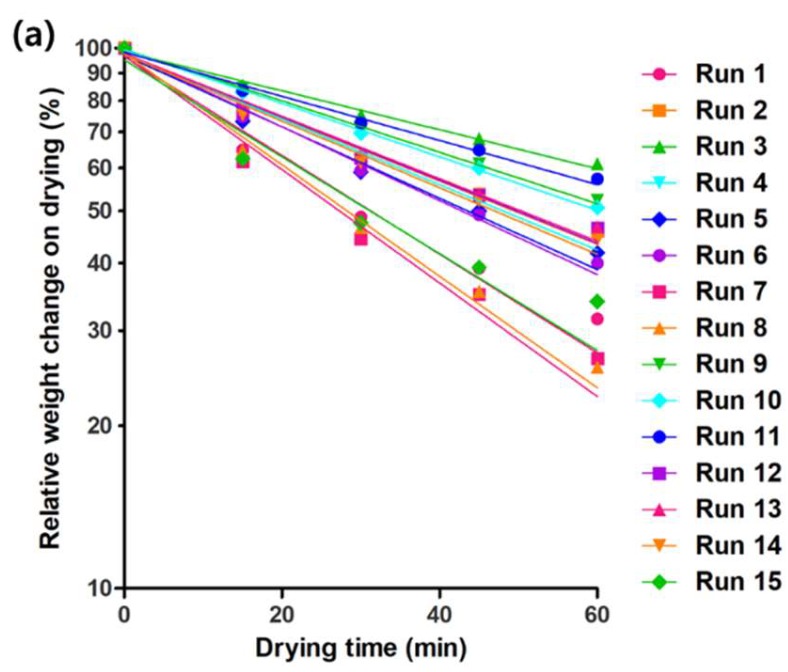

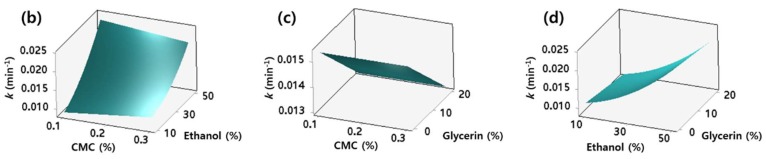
The weight loss of the hydrosol on drying (**a**). The best-fit straight line (first-order kinetics) for each run is shown together. Response surface plots exhibiting the effect of (**b**) CMC (*x*_1_) and ethanol (*x*_2_); (**c**) CMC (*x*_1_) and glycerin (*x*_3_); and (**d**) ethanol (*x*_2_) and glycerin (*x*_3_) on drying rate constant (*k*; *y*_2_). The old values of CMC, ethanol, and glycerin were set at 0.2%, 30%, and 10%, respectively.

The weight loss of the hydrosol on drying is shown in Figure 4a, and the drying rate constant (*k*) values were successfully calculated by fitting the first-order model to these data (*R*^2^ > 0.951; Table 1). The *k*-values ranged from 0.0083–0.0242 min^−1^, and the reduced model equation for *k* (*y*_2_) in coded units is as below:

*y*_2_ = 0.0142 + 0.0062·*x*_2_ − 0.00114·*x*_3_ + 0.00202·*x*_2_·*x*_2_ − 0.00118·*x*_1_·*x*_2_ (2)

which consists only of the terms having a significant effect on the *k*-values (*p* < 0.05; Table 2). The positive coefficients of *x*_2_ and *x*_2_·*x*_2_ terms imply that the *k*-values increased as the concentration of ethanol raised, whereas the negative coefficient of *x*_3_ term clearly showed the inhibitory effect of the glycerin on drying. The actual, adjusted, and predicted *R*^2^ values of this model were 0.9839, 0.9775, and 0.9659, respectively, supporting that the model can explain the *k*-values more than 98% without being over-fitted. The response surface plots exhibiting the relationship between the independent variables and the *k*-values of the hydrosol are presented in Figure 4b–d. Noteworthy is that although the interaction term, *x*_1_·*x*_2_, has the negative coefficient value, the overall effect of the ethanol concentration on the *k*-values was positive under the experimental conditions. 

### 2.5. Optimization of AdP Hydrosol Based on Composite Desirability

**Figure 5:**
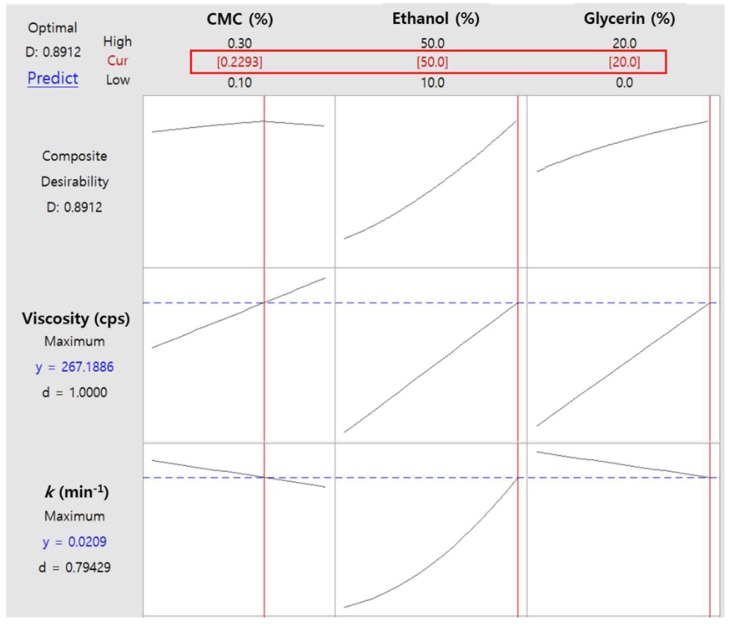
Optimization plots for viscosity and drying rate constant (*k*). The composition of CMC, ethanol, and glycerin exhibiting the maximum composite desirability is presented in the red box.

The optimization plots exhibiting the desirability functions are shown in Figure 5. The maximum composite desirability (*D*) of 0.8912 was obtained at the CMC, ethanol, and glycerin concentrations of 0.229% (*w*/*v*), 50% (*v*/*v*), and 20% (*v*/*v*), respectively. The *D*-value close to 1 suggests that both the viscosity and *k* achieved the favorable (i.e., maximized) results as a whole. The individual desirability values for viscosity (*d*_1_) and *k* (*d*_2_) were 1.0000 and 0.79429, respectively, implying the above composition of hydrosol is more effective in maximizing viscosity than *k*. Of note, although the concentrations of CMC and glycerin exhibited opposite effects on viscosity and *k*, only the CMC concentration term displayed maximum point in the *D*-function. The ethanol concentration term showed only a positive effect under our experimental condition. 

### 2.6. Artificial Skin Deposition Study 

**Figure 6:**
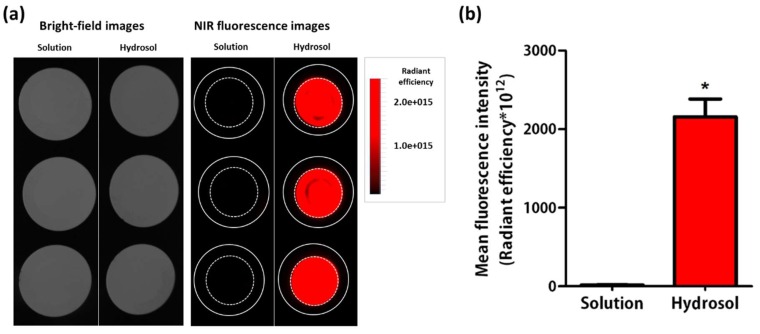
In vitro artificial skin deposition of the fluorescent dye-tagged AdP: (**a**) NIR image of STM membranes and (**b**) their mean fluorescence intensity values after 8 h of incubation are shown. The dashed circle means the effective surface area (1.76 cm^2^) of the Keshary-Chien cell. Data are presented as the mean ± SD (*n* = 3). * *p* < 0.05, significantly different from each other.

The Strat-M membrane (STM; artificial skin) deposition of the fluorescent dye-tagged FA-AdP hydrosol was monitored by near-infrared fluorescence (NIR) imaging (Figure 6). Based on the results of the optimization studies, the labeled FA-AdP loaded hydrosol containing CMC, ethanol, and glycerin at concentrations of 0.229% (*w*/*v*), 50% (*v*/*v*), and 20% (*v*/*v*) was selected as the final formulation. At 8 h incubation, the mean fluorescence intensity of the hydrosol treated STM was 127-fold higher than that of the free FA-AdP (i.e., the solution in double-deionized water) treated STM (*p* < 0.05). However, the coefficient of variation (CV) values were comparable between the two groups (10.6% and 14.8% for FA-AdP-loaded hydrosol and free FA-AdP, respectively), supporting the reproducibility of the dramatic enhancing effect of the developed hydrosol. 

## 3. Discussion

AdP was dissected to determine the functional domain. Fibroblast-activating domain of AdP (FA-AdP, sh-oligopeptide 84) and melanocyte-inhibiting domain of AdP (MI-AdP, sh-oligopeptide 91 SP) were proved as 14–28 and 18–32 residues of AIMP1, respectively. Despite the 66% homology between their sequences, these two peptides showed different functions in skin-associated cells, which could be explained by the difference in secondary/tertiary structures or interaction partners. Characteristic three-dimensional structures enable even the high homology peptides to have distinct biological activities, providing different interaction interfaces [35]. Meanwhile, FA-AdP and MI-AdP are 15-amino acid (aa) length peptides, which are much shorter than AdP (41-aa length peptide). In general, truncated peptides have advantages such as the feasibility of simple and cost-effective synthesis and enhancement in skin penetration, suggesting FA-AdP and MI-AdP are attractive cosmeceutical ingredients.

However, according to the previous study, these peptides need to be delivered to the dermis—where the fibroblasts reside in—to exert skin anti-aging activity [13]. Thus, hydrosol-type formulations were developed to address this delivery issue. Ethanol and glycerol were employed as the main components of the truncated AdP-loaded hydrosol. Ethanol is a commonly used penetration enhancer in the cosmetic industry, which can improve the peptide delivery to the skin by multiple mechanisms. Ethanol can alter the physical state of the skin tissue or even can extract some of the lipids composing the SC layer, resulting in the improvement of peptide flux through the skin [36]. Moreover, ethanol may increase the peptide concentration after administration due to its rapid loss by evaporation or skin absorption. The consequent supersaturation of peptide may provide a higher skin delivery potential [26]. Ethanol can also directly drag the peptide into the skin during its penetration [37]. Although ethanol concentrations up to 80% can be used without skin irritation concerns [38], the precipitation of CMC at high concentrations of ethanol limited its maximum content in the hydrosol to 50% in this study. Glycerin can also improve the peptide delivery efficiency, of which underlying mechanism is the hydration of the SC layer [39]. The skin hydration can augment the percutaneous absorption of the hydrophilic compounds, including peptides [26].

Moreover, the viscosity and *k*-values may also affect the peptide delivery efficiency, as well as the convenience of application [40,41]. More specifically, higher viscosity is closely related to the longer skin retention of the hydrosol, and faster drying rate guarantees the rapid development of the peptide concentration gradient between the hydrosol and the skin [40,41]. According to Fick’s first law of diffusion, both of these properties need to be maximized to obtain the highest flux of peptide. Thus, the viscosity and *k*-values were regarded as critical quality attributes (CQAs) in this study. To achieve adequate viscosity, CMC was added to the hydrosol as a thickening agent. However, as CMC precipitates in the presence of a high concentration of ethanol, the CMC was not added at more than 0.3%. Interestingly, even under this low concentration, CMC exhibited a negative effect on the drying rate in our preliminary study (data not shown), suggesting the necessity of a systematic approach to achieving its optimized content. Therefore, BBD was adopted to evaluate the relationship between the compositions and CQAs of the hydrosol more thoroughly. Based on this relationship, the optimized composition that shows the maximized *D*-value was obtained. 

The optimized hydrosol was evaluated for its AdPs delivery efficiency to the skin. However, the employment of conventional study models that use animal skin is discouraged when testing cosmeceuticals [42]. As an alternative of animal skin, STM, a synthetic membrane, was adopted, which shares a similar structure with the human skin. A tight top layer coated with lipids resembles the SC. Underneath the top layer, the two layers of polyethersulfone (PES) are supported by the polyolefin non-woven matrix. These structures mimic the dermis and subcutaneous tissue, respectively [43]. After the artificial skin deposition study, the STM samples were evaluated using the NIR imaging instrument to quantify the FA-AdP deposition. NIR imaging provides the information on the total extent of peptide deposition on STM almost instantaneously. Moreover, as this technique does not require destructive sample pretreatments, such as homogenization, extraction, and concentration, the fluorescence intensity of the intact samples can be visualized with spatial information. The observed dramatic enhancement of FA-AdP deposition in the artificial skin suggests that the developed hydrosol could be used as a promising topical AdPs delivery system for skin anti-aging.

## 4. Materials and Methods

### 4.1. Cell Culture and Reagents

Foreskin fibroblasts (HFF-1), keratinocytes (HaCaT), and melanocytes (B16F10) were cultured in DMEM medium supplemented with 10% fetal bovine serum (FBS) and antibiotics (100 U/mL penicillin and 100 μg/mL streptomycin). THP-1 cells were cultured in RPMI 1640 medium supplemented with 10% FBS and antibiotics (100 U/mL penicillin and 100 μg/mL streptomycin). Cells were cultured at 37 °C in a humidified atmosphere at 5% CO_2_. All peptides were synthesized by the solid-phase synthesis method, as previously reported by our group [13]. Blanose™ sodium carboxymethylcellulose 7LP EP (CMC; 90.5 kDa) was purchased from Ashland (Schaffhausen, Switzerland). Glycerin and ethanol were obtained from Samchun Chemical Co., Ltd. (Seoul, Korea). Alexa Fluor™ 647 NHS ester was purchased from Thermo Fisher Scientific (Waltham, MA, USA). Strat-M membrane (STM) was obtained from EMD Millipore Co. (Billerica, MA, USA). Phosphate buffered saline (PBS) was purchased from Lonza Group Ltd. (Basel, Switzerland). All other reagents were of analytical grade and were purchased from commercial sources.

### 4.2. Collagen ELISA

Foreskin fibroblast cells (HFF-1; 5 × 10^4^ cells/well) were seeded at a 24-well culture plate. Before peptide treatment, cells were pre-incubated with serum-free DMEM media for 6 h. Cells were treated with AdP, hEGF (Sigma-Aldrich, St. Louis, MO, USA) and fragments of AdP for 24 h. After 24 h treatment, media were harvested for collagen ELISA assay. Procollagen type I in the culture media was determined by Procollagen type I ELISA kit (Takara, Kusatsu, Japan) in accordance with the manufacturer instructions. Procollagen type I amount in the media was normalized by the Procollagen type I amount of control group.

### 4.3. Proliferation Assay

Foreskin fibroblast cells (HFF-1; 3 × 10^3^ cells/well) were seeded into 96-well plate and cultivated. Cells were replaced with serum-free media and then treated with AdP, hEGF, and fragments of AdP for 48 h. Cell proliferation was determined CCK-8 assay (Dojindo, Kumamoto, Japan) in accordance with the manufacturer instructions. Proliferation was normalized by the proliferation of control group.

### 4.4. Measurement of Melanin 

Melanocytes (B16F10; 1 × 10^5^ cells/well) were seeded into 6-well plate and cultivated. To induce melanin synthesis, α-MSH (0.5 μM), melanin inducer, was used. AdP and fragments of AdP were treated with α-MSH (0.5 μM) for 48 h. Intracellular melanin and tyrosinase activity were monitored as previously reported [13]. Briefly, melanocytes (1 × 10^5^ cells,) were lysed with 1 N NaOH at 70 °C for 1 h. Intracellular melanin was determined in lysed samples by 490 nm wavelength absorbance using microplate reader (Synergy 2, BioTek, Winooski, VM, USA). 

### 4.5. Cytotoxicity and Immunogenicity Tests 

The safety of FA-AdP and MI-AdP was verified in terms of cytotoxicity and immunogenicity. To test the cytotoxicity of the peptides, keratinocytes (HaCaT; 3 × 10^3^ cells/well) were seeded into 96-well plate and cultivated. Cells were incubated with doxorubicin (Dox, 10 μM), AdP (100 μg/mL), FA-AdP (10 or 100 μg/mL), or MI-AdP (10 or 100 μg/mL). After 24 h incubation, cytotoxicity was determined by CCK-8 assay (Dojindo) according to the manufacturer’s manual. The immunogenicity of the peptides was evaluated by performing TNF-α ELISA. THP-1 cells (5 × 10^4^ cells/well) were seeded on a 24-well plate. Cells were pre-incubated with 1% FBS containing media for 6 h. After pre-incubation, cells were treated with 10 ng/mL LPS, AdP, and fragments of AdP. After 12 h treatment, conditioned media were harvested for TNF- α ELISA assay. TNF-α amount in conditioned media was assessed by human TNF-α ELISA kit (BD Bioscience, San Jose, CA, USA) in accordance with the manufacturer instructions. TNF-α amount in conditioned media was normalized by TNF-α amount of control group.

### 4.6. Preparation of FA-AdP-Loaded Hydrosol

FA-AdP hydrosol formulations were prepared using CMC, glycerin, and ethanol. CMC was dissolved in hot double-deionized water (DDW; 80 °C) by continuous stirring at 700 rpm for 3 h to prepare 3% (*w*/*v*) working solution. Glycerin and ethanol were mixed with the CMC solution using homogenizer (Ultra-Turrax T-25, IKA Works GmbH & Co. KG, Staufen, Germany) at 14,000 rpm for 5 min. FA-AdP dissolved in DDW was added to the mixture to produce the final formulation with FA-AdP concentration of 20 µM. 

### 4.7. Experimental Design for Hydrosol Optimization 

The Box-Behnken design (BBD) was employed to optimize the physicochemical properties of FA-AdP hydrosol. The concentrations of CMC (*x*_1_), ethanol (*x*_2_), and glycerin (*x*_3_) were chosen as independent variables. The effects of these factors on the viscosity (*y*_1_) and drying rate constant (*y*_2_) of hydrosol were evaluated at three levels. Including three replicates in the center, 15 experiments were performed. The experimental combinations are presented in Table 1. The best-fit quadratic models were generated using Minitab^®^ 18.1 (Minitab Inc., State College, PA, USA) to investigate linear, quadratic, and interaction effects of the three factors. Only the variables with a *p*-value < 0.05 were considered significant and included in the model. Based on the results of randomized experimental runs, the 3-dimensional surface plots were obtained. The optimization plots showing desirability functions were generated, and the combination of independent variables exhibiting the maximum composite desirability (*D*) was estimated. The *D*-value was obtained as the geometric mean of the individual desirability (*d*) using below formula:

*D* = (*d*_1_·*d*_2_)^1/2^ (3)

where *d*_1_ and *d*_2_ represent the individual desirability of viscosity and drying rate constant of the hydrosol, respectively, and no weighting factor was applied.

### 4.8. Measurement of Viscosity and Drying Rate

The viscosity of the FA-AdP hydrosol was measured by using DV-II+Pro viscometer equipped with an LV-1 spindle (Ametek Brookfield, Middleborough, MA, USA). The measurement was made between 50–60% of the instrument torque scale at 25 °C. To evaluate the drying rate, aliquots (200 μL) of the developed formulations were loaded in dishes (surface area: 63.6 mm^2^) of a known weight and incubated in a dry oven (32 °C). Their weight changes were monitored at 0, 15, 30, 45, and 60 min. The drying rate constant (*k*; min^−1^) was calculated by using the first-order correlation between the weight change on drying and elapsed time. The regression lines were plotted according to the following equation:

*A*_t_ = *A*_0_·e^−*k*t^ (4)

where *A*_0_ and *A*_t_ represent the weights of the hydrosol at time 0 and t (min), respectively.

### 4.9. Evaluation of Artificial Skin Deposition by Near-Infrared Fluorescence Imaging

The artificial skin deposition study was conducted using NIR imaging. FA-AdP (4 mg) was dissolved in 0.1 M sodium bicarbonate buffer (1 mL, pH 8.3) and slowly mixed with Alexa Fluor™ 647 NHS ester (50 μg). The mixture was incubated at room temperature for 2 h. Fluorescence-labeled FA-AdP was purified by using a PD-10 desalting column (GE Healthcare, Chicago, IL, USA), and its concentration was measured by bicinchoninic acid protein assay kit (Thermo Fisher Scientific, Waltham, MS, USA) according to the manufacturer’s protocol.

Fluorescence-labeled FA-AdP-loaded hydrosol was prepared according to the same method in Section 4.6. The composition of the hydrosol showing the highest composite desirability was selected as the optimized formulation for the skin delivery study. The concentration of fluorescence-labeled FA-AdP in the hydrosol was 20 µM. An aliquot (1 mL) of the labeled FA-AdP hydrosol or labeled FA-AdP dissolved in DDW (20 µM, control group) was applied to the upper side of STM clamped between the donor and receptor cells of Keshary-Chien diffusion cell (FCDV-15 Diffusion Cell Drive Console; Labfine, Anyang, Korea). The diffusion cell area was 1.76 cm^2^, and the experiment was performed at 32 °C under dark condition. After 8 h of incubation, the STM was collected and washed with an excess amount of DDW for 5 times. The NIR intensity of the artificial skin samples was measured by VISQUE ^®^ InVivo Smart (Vieworks, Anyang, Korea). 

### 4.10. Statistics

The statistical analysis (unpaired two-tailed Student’s *t*-test) was performed using SPSS statistics software (Version 21.0; IBM Corp, NY, USA). All results are presented as the means ± standard deviation (SD), and the *p*-value of less than 0.05 was considered statistically significant.

## Figures and Tables

**Table 1:** Experimental combinations of independent variables and observed responses in Box-Behnken design.

Run Order	Independent Variables			Dependent Variables	
	*x*_1_	*x*_2_	*x*_3_	*y*_1_	*y*_2_
	CMC (%, *w*/*v*)	Ethanol (%, *v*/*v*)	Glycerin (%, *v*/*v*)	Viscosity (cps)	Drying Rate Constant (1/min)
1	0.3	50	10	261.1	0.0210
2	0.1	30	20	173.7	0.0135
3	0.2	10	20	158.7	0.0083
4	0.3	30	0	171.0	0.0139
5	0.2	30	10	165.0	0.0152
6	0.1	30	0	137.1	0.0157
7	0.1	50	10	173.7	0.0242
8	0.2	50	0	146.4	0.0238
9	0.3	10	10	188.1	0.0110
10	0.2	10	0	116.1	0.0114
11	0.1	10	10	122.1	0.0094
12	0.2	30	10	173.7	0.0135
13	0.3	30	20	212.4	0.0133
14	0.2	30	10	178.5	0.0142
15	0.2	50	20	267.1	0.0206

**Table 2:** Response surface regression of viscosity (*y*_1_) and drying rate constant (*y*_2_).

Responses	Sources	DF ^1^	SS ^1^	MS ^1^	*F*-Value	*p*-Value
*y*_1_	Model	4	23853.5	5963.4	23.78	<0.001
	*x*_1_	1	6384.5	6384.5	25.46	0.001
	*x*_2_	1	8665.9	8665.9	34.56	<0.001
	*x*_3_	1	7278.2	7278.2	29.02	<0.001
	*x*_2_·*x*_3_	1	1524.9	1524.9	6.08	0.033
	Error	10	2507.6	250.8		
	Total	14	26361.1			
*y*_2_	Model	4	0.000338	0.000085	152.99	<0.001
	*x*_2_	1	0.000307	0.000307	555.63	<0.001
	*x*_3_	1	0.00001	0.00001	18.86	0.001
	*x*_2_·*x*_2_	1	0.000015	0.000015	27.44	<0.001
	*x*_1_·*x*_2_	1	0.000006	0.000006	10.04	0.01
	Error	10	0.000006	0.000001		
	Total	14	0.000344			

^1^ Degrees of freedom (DF); adjusted sum of squares (SS); adjusted mean squares (MS).

## References

[1] Ganceviciene R., Liakou A.I., Theodoridis A., Makrantonaki E., Zouboulis C.C. (2012). Skin anti-aging strategies. Dermato-endocrinology.

[2] Pena Ferreira M.R., Costa P.C., Bahia F.M. (2010). Efficacy of anti-wrinkle products in skin surface appearance: a comparative study using non-invasive methods. Skin. Res. Technol..

[3] Hynes R.O. (2009). The extracellular matrix: not just pretty fibrils. Science.

[4] Frantz C., Stewart K.M., Weaver V.M. (2010). The extracellular matrix at a glance. J. Cell Sci..

[5] Clause K.C., Barker T.H. (2013). Extracellular matrix signaling in morphogenesis and repair. Curr. Opin. Biotechnol..

[6] Malerich S., Berson D. (2014). Next generation cosmeceuticals: the latest in peptides, growth factors, cytokines, and stem cells. Dermatol. clin..

[7] Uitto J. (2008). The role of elastin and collagen in cutaneous aging: intrinsic aging versus photoexposure. J. Drugs Dermato..

[8] Fligiel S.E., Varani J., Datta S.C., Kang S., Fisher G.J., Voorhees J.J. (2003). Collagen degradation in aged/photodamaged skin in vivo and after exposure to matrix metalloproteinase-1 in vitro. J. Investig. Dermatol..

[9] Varani J., Perone P., Fligiel S.E., Fisher G.J., Voorhees J.J. (2002). Inhibition of type I procollagen production in photodamage: correlation between presence of high molecular weight collagen fragments and reduced procollagen synthesis. J. Invest. Dermatol..

[10] Carsberg C.J., Warenius H.M., Friedmann P.S. (1994). Ultraviolet radiation-induced melanogenesis in human melanocytes. Effects of modulating protein kinase C. J. Cell Sci..

[11] Brenner M., Hearing V.J. (2008). The protective role of melanin against UV damage in human skin. Photochem. Photobiol..

[12] Lintner K., Mas-Chamberlin C., Mondon P., Peschard O., Lamy L. (2009). Cosmeceuticals and active ingredients. Clin. Dermatol..

[13] Kim J., Kang S., Kwon H., Moon H., Park M.C. (2019). Dual functional bioactive-peptide, AIMP1-derived peptide (AdP), for anti-aging. J. Cosmet. Dermatol..

[14] Kim S., You S., Hwang D. (2011). Aminoacyl-tRNA synthetases and tumorigenesis: more than housekeeping. Nat. Rev. Cancer.

[15] Lee S.W., Kim G., Kim S. (2008). Aminoacyl-tRNA synthetase-interacting multi-functional protein 1/p43: an emerging therapeutic protein working at systems level. Expert Opin. Drug Discov..

[16] Helfrich Y.R., Sachs D.L., Voorhees J.J. (2008). Overview of skin aging and photoaging. Dermatol. Nurs..

[17] Park S.G., Shin H., Shin Y.K., Lee Y., Choi E.C., Park B.J., Kim S. (2005). The novel cytokine p43 stimulates dermal fibroblast proliferation and wound repair. Am. J. Pathol..

[18] Han J.M., Park S.G., Lee Y., Kim S. (2006). Structural separation of different extracellular activities in aminoacyl-tRNA synthetase-interacting multi-functional protein, p43/AIMP1. Biochem. Biophys. Res. Commun..

[19] Witting M., Obst K., Friess W., Hedtrich S. (2015). Recent advances in topical delivery of proteins and peptides mediated by soft matter nanocarriers. Biotechnol. Adv..

[20] Kang N.W., Kim M.H., Sohn S.Y., Kim K.T., Park J.H., Lee S.Y., Lee J.Y., Kim D.D. (2018). Curcumin-loaded lipid-hybridized cellulose nanofiber film ameliorates imiquimod-induced psoriasis-like dermatitis in mice. Biomaterials.

[21] Aufenvenne K., Larcher F., Hausser I., Duarte B., Oji V., Nikolenko H., Del Rio M., Dathe M., Traupe H. (2013). Topical enzyme-replacement therapy restores transglutaminase 1 activity and corrects architecture of transglutaminase-1-deficient skin grafts. Am. J. Hum. Genet..

[22] Goebel A.S., Schmaus G., Neubert R.H., Wohlrab J. (2012). Dermal peptide delivery using enhancer molecules and colloidal carrier systems--part I: carnosine. Skin Pharmacol. Physiol..

[23] Prausnitz M.R., Mitragotri S., Langer R. (2004). Current status and future potential of transdermal drug delivery. Nat. Rev. Drug Discov..

[24] Denet A.R., Vanbever R., Preat V. (2004). Skin electroporation for transdermal and topical delivery. Adv. Drug Deliv. Rev..

[25] Prausnitz M.R. (2004). Microneedles for transdermal drug delivery. Adv. Drug Deliv. Rev..

[26] Williams A.C., Barry B.W. (2004). Penetration enhancers. Adv. Drug Deliv. Rev..

[27] Magnusson B.M., Runn P. (1999). Effect of penetration enhancers on the permeation of the thyrotropin releasing hormone analogue pGlu-3-methyl-His-Pro amide through human epidermis. Int. J. Pharm..

[28] Butun S., Ince F.G., Erdugan H., Sahiner N. (2011). One-step fabrication of biocompatible carboxymethyl cellulose polymeric particles for drug delivery systems. Carbohydr. Polym..

[29] Pushpamalar V., Langford S.J., Ahmad M., Lim Y.Y. (2006). Optimization of reaction conditions for preparing carboxymethyl cellulose from sago waste. Carbohydr. Polym..

[30] Kim J.-S., Kim K.-T., Park J.-H., Lee J.-Y., Kim M., Min H.G., Moon I.-H., Choi C.-Y., Kim B.H., Kim D.-D. (2018). Coacervate microcapsules of vitamin U optimized by central composite design (CCD). J. Pharm. Investig..

[31] Ahmed Z.Z., Khan F.N., Shaikh D.A. (2018). Reverse engineering and formulation by QBD of olopatadine hydrochloride ophthalmic solution. J. Pharm. Investig..

[32] Navamanisubramanian R., Nerella R., Duraipandian C., Seetharaman S. (2018). Quality by design approach for optimization of repaglinide buccal tablets using Box-Behnken Design. Future J. Pharm. Sci..

[33] Prabhakaran D., Basha C.A., Kannadasan T., Aravinthan P. (2010). Removal of hydroquinone from water by electrocoagulation using flow cell and optimization by response surface methodology. J. Environ. Sci. Health A.

[34] Natrella M. (2010). NIST/SEMATECH e-Handbook of Statistical Methods.

[35] Amir A.I., van Rosmalen M., Mayer G., Lebendiker M., Danieli T., Friedler A. (2015). Highly homologous proteins exert opposite biological activities by using different interaction interfaces. Sci. Rep..

[36] Megrab N.A., Williams A.C., Barry B.W. (1995). Oestradiol permeation across human skin, silastic and snake skin membranes: the effects of ethanol/water co-solvent systems. Int. J. Pharm..

[37] Morimoto H., Wada Y., Seki T., Sugibayashi K. (2002). In vitro skin permeation of morphine hydrochloride during the finite application of penetration-enhancing system containing water, ethanol and l-menthol. Biol. Pharm. Bull..

[38] Löffler H., Kampf G., Schmermund D., Maibach H. (2007). How irritant is alcohol?. Br. J. Dermatol..

[39] Maibach H.I., Lodén M. (2000). Dry Skin and Moisturizers: Chemistry and Function.

[40] Chang R.-K., Raw A., Lionberger R., Yu L. (2012). Generic development of topical dermatologic products: formulation development, process development, and testing of topical dermatologic products. AAPS J..

[41] Lu W., Luo H., Zhu Z., Wu Y., Luo J., Wang H. (2014). Preparation and the biopharmaceutical evaluation for the metered dose transdermal spray of dexketoprofen. J. Drug Deliv..

[42] Adler S., Basketter D., Creton S., Pelkonen O., van Benthem J., Zuang V., Andersen K.E., Angers-Loustau A., Aptula A., Bal-Price A. (2011). Alternative (non-animal) methods for cosmetics testing: current status and future prospects—2010. Arch. Toxicol..

[43] Haq A., Goodyear B., Ameen D., Joshi V., Michniak-Kohn B. (2018). Strat-M® synthetic membrane: Permeability comparison to human cadaver skin. Int. J. Pharm..

